# The genome sequence of the grizzled skipper, *Pyrgus malvae *(Linnaeus, 1758)

**DOI:** 10.12688/wellcomeopenres.17806.1

**Published:** 2022-03-29

**Authors:** Alex Hayward, Roger Vila, Konrad Lohse, Dominik Laetsch, Darwin Tree of Life Barcoding collective, Wellcome Sanger Institute Tree of Life programme, Wellcome Sanger Institute Scientific Operations: DNA Pipelines collective, Tree of Life Core Informatics collective, Darwin Tree of Life Consortium

**Affiliations:** 1 College of Life and Environmental Sciences, Department of Biosciences, University of Exeter, Penryn, UK; 2 Institut de Biologia Evolutiva (CSIC - Universitat Pompeu Fabra), Barcelona, Spain; 3 Institute of Evolutionary Biology, University of Edinburgh, Edinburgh, UK

**Keywords:** Pyrgus malvae, grizzled skipper, genome sequence, chromosomal, Lepidoptera

## Abstract

We present a genome assembly from an individual male *Pyrgus malvae *(the grizzled skipper; Arthropoda; Insecta; Lepidoptera; Hesperiidae). The genome sequence is 725 megabases in span. The majority (99.97%) of the assembly is scaffolded into 31 chromosomal pseudomolecules, with the Z sex chromosome assembled.

## Species taxonomy

Eukaryota; Metazoa; Ecdysozoa; Arthropoda; Hexapoda; Insecta; Pterygota; Neoptera; Endopterygota; Lepidoptera; Glossata; Ditrysia; Papilionoidea; Hesperiidae; Pyrginae; *Pyrgus*; *Pyrgus malvae* (Linnaeus, 1758) (NCBI:txid218760).

## Background

The grizzled skipper, *Pyrgus malvae*, is a small butterfly, characteristic of chalk downland and woodland clearings, and other grassland habitats. Not to be confused with the term ‘grisly’ (i.e. extremely unpleasant or gruesome), *P. malvae* gets its common name from the tufts of long grey hair that cover its body and inner wings. Its wings bear a striking black and white checkerboard pattern, with alternating black and white stripes on the wing fringes and antennae. Notoriously difficult to follow, *P. malvae* has a fast and darting, low flight pattern. *Pyrgus malvae* is found throughout Europe, except for northern Scandinavia, several Mediterranean Islands and Iberia, southern France and Italy (where it is replaced by its sister species *P. malvoides*), with a range that extends eastwards across temperate Asia to Northern China and Korea ( Tolman & Lewington, 2008). In the UK the species is found mainly in central and southern England, with a patchy distribution in Wales and the southwest. *P. malvae* typically exists in small populations (<100 adults) that are thought to form metapopulations across its range ( Asher *et al.*, 2001).

In the UK, *P. malvae* typically emerges in April and flies until June, although the date of first emergence is advancing, and in warm years may occur as early as March . It is univoltine in northern Europe and at higher altitudes, but is bivoltine elsewhere, and in the north it may be bivoltine when weather conditions are particularly favourable ( Asher *et al.*, 2001).

*Pyrgus malvae* larvae feed on a variety of host plants in the Rosaceae family, particularly agrimony ( *Agrimonia eupatoria*), creeping cinquefoil ( *Potentilla reptans*) and wild strawberry ( *Fragaria vesca*) ( Asher *et al.*, 2001). When fully-grown, the larva constructs a cocoon at the base of low vegetation, where it overwinters as a pupa. Adults feed on a wide variety of nectar sources, including Bird’s foot trefoil ( *Lotus corniculatus*), bugle ( *Ajuga reptans*), buttercup ( *Ranunculus* species), daisy ( *Bellis perennis*), and dandelion ( *Taraxacum officinale*). Males are territorial, and exhibit either perching or patrolling behaviour according to habitat type ( Brereton *et al*., 1998), and have two scent organs: the forewing costal fold and tibial tufts composed of specialised setae on the hind leg, which appear to be used to waft pheromones towards the female during courtship ( Hernández-Roldán *et al.*, 2014). Eggs are laid singly on the leaf underside of larval host plants, with the majority deposited on short vegetation in locations with a favourably warm microclimate and/or elevated nutritional content ( Brereton *et al*., 1998).

Populations of *P. malvae* in the UK have declined markedly in the twentieth century ( Brereton *et al*., 1998) and the species is a conservation priority in the UK ( Brig, 2007). Encouragingly, *P. malvae* appears to be positively associated with grazed vegetation, and implementing grazing in habitat restoration regimes may offer a means to help reverse population declines ( Wallis De Vries & Raemakers, 2001). At European level this species is listed as Least Concern in the IUCN Red List ( Van Swaay *et al.*, 2010). *Pyrgus malvae* has been reported as having 33 ( Bigger, 1960; England) and 31 ( Federley, 1938; Finland) chromosome pairs. The assembly described herein contains 31 chromosome pairs.

## Genome sequence report

**Figure 1.**
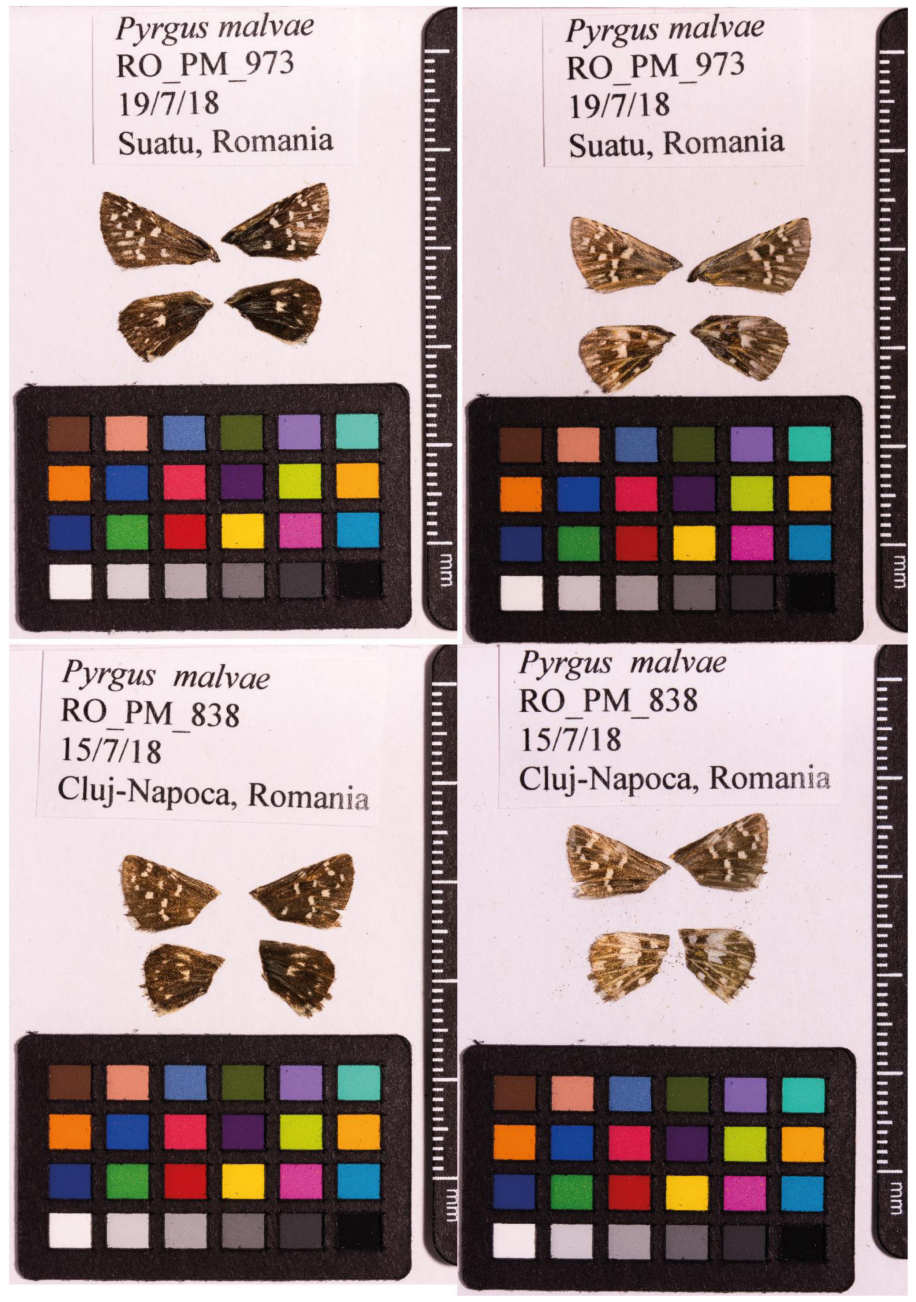
Fore and hind wings of the *Pyrgus malvae* specimen from which the genome was sequenced. Top: Dorsal (left) and ventral (right) surface view of wings from specimen RO_PM_973 (ilPyrMalv3) from Suatu, Romania, used to generate Pacific Biosciences and 10X genomics data. Bottom: Dorsal (left) and ventral (right) surface view of wings from specimen RO_PM_838 (ilPyrMalv2) from Cluj-Napoca, Romania, used to generate Hi-C data.

The genome was sequenced from a single male *P. malvae* ( Figure 1) collected from Suatu, Cluj County, Romania (latitude 46.7648, longitude 23.9845). A total of 69-fold coverage in Pacific Biosciences single-molecule circular consensus (HiFi) long reads and 49-fold coverage in 10X Genomics read clouds were generated. Primary assembly contigs were scaffolded with chromosome conformation Hi-C data. Manual assembly curation corrected 2 missing/misjoins and removed 1 haplotypic duplication, reducing the assembly length by 0.01% and the scaffold number by 7.69%.

**Figure 2.**
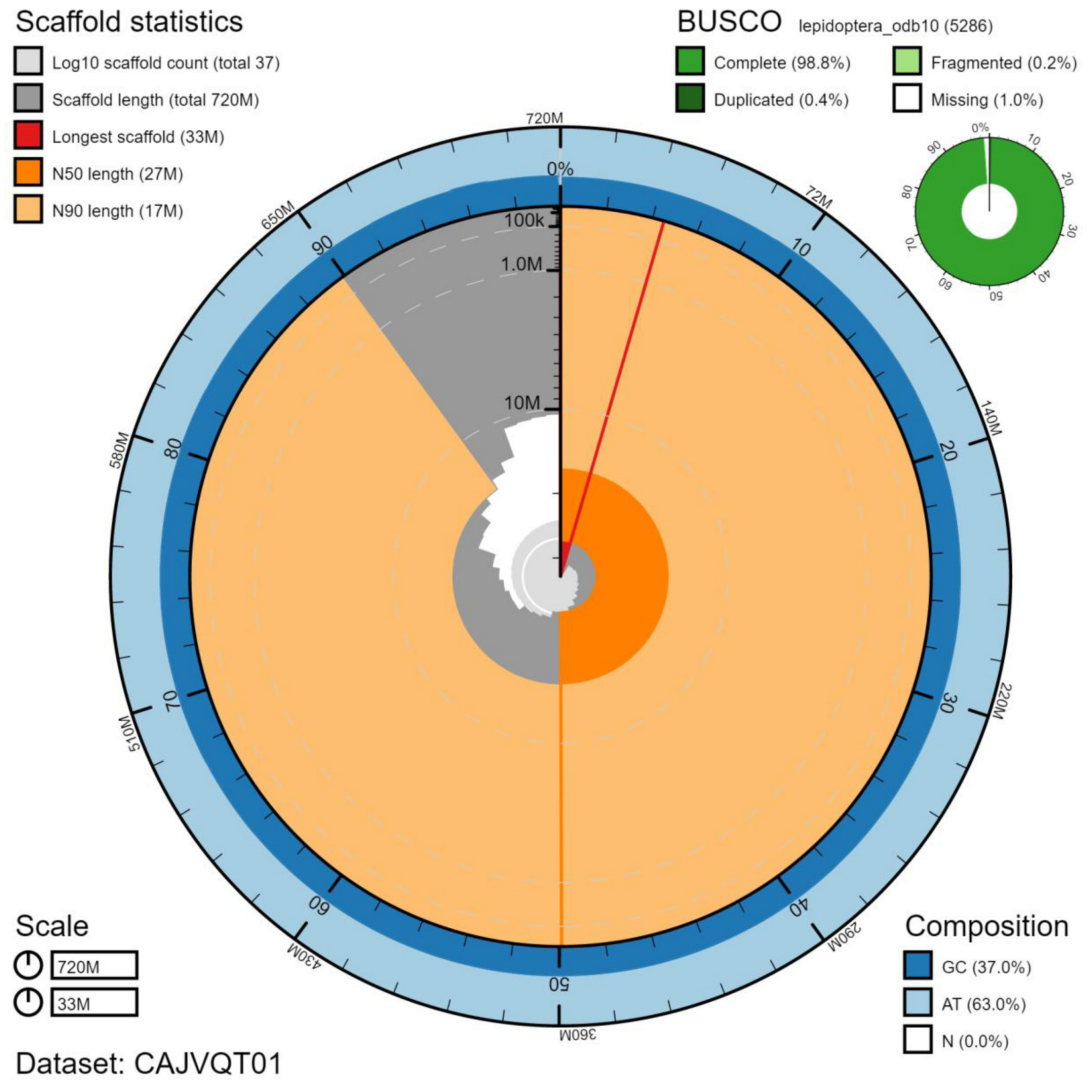
Genome assembly of *Pyrgus malvae*, ilPyrMalv3.1: metrics. The BlobToolKit Snailplot shows N50 metrics and BUSCO gene completeness. The main plot is divided into 1,000 size-ordered bins around the circumference with each bin representing 0.1% of the 724,649,524 bp assembly. The distribution of chromosome lengths is shown in dark grey with the plot radius scaled to the longest chromosome present in the assembly (33,217,309 bp, shown in red). Orange and pale-orange arcs show the N50 and N90 chromosome lengths (26,976,370 and 16,663,010 bp), respectively. The pale grey spiral shows the cumulative chromosome count on a log scale with white scale lines showing successive orders of magnitude. The blue and pale-blue area around the outside of the plot shows the distribution of GC, AT and N percentages in the same bins as the inner plot. A summary of complete, fragmented, duplicated and missing BUSCO genes in the lepidoptera_odb10 set is shown in the top right. An interactive version of this figure is available at https://blobtoolkit.genomehubs.org/view/ilPyrMalv3.1/dataset/CAJVQT01/snail.

The final assembly has a total length of 725 Mb in 36 sequence scaffolds with a scaffold N50 of 27.0 Mb ( Table 1). The majority, 99.97%, of assembly sequence was assigned to 31 chromosomal-level scaffolds, representing 30 autosomes (numbered by sequence length), and the Z sex chromosome ( Figure 2– Figure 5; Table 2). The assembly has a BUSCO v5.1.2 ( Manni *et al.*, 2021) completeness of 98.8% (single 98.3%, duplicated 0.4%) using the lepidoptera_odb10 reference set (n=5286). While not fully phased, the assembly deposited is of one haplotype. Contigs corresponding to the second haplotype have also been deposited.

**Table 1.** Genome data for *Pyrgus malvae*, ilPyrMalv3.1.

*Project accession data*	
Assembly identifier	ilPyrMalv3.1
Species	*Pyrgus malvae*
Specimen	ilPyrMalv3 (genome assembly); ilPyrMalv2 (Hi-C); ilPyrMalv1 (RNA-Seq)
NCBI taxonomy ID	NCBI:txid111923
BioProject	PRJEB46857
BioSample ID	SAMEA7523296
Isolate information	Male, whole organism (ilPyrMalv3); unknown sex, whole organisms (ilPyrMalv1, ilPyrMalv2)
*Raw data accessions*	
PacificBiosciences SEQUEL II	ERR6606794-ERR6606796
10X Genomics Illumina	ERR6363273-ERR6363276
Hi-C Illumina	ERR6363278
Illumina polyA RNA-Seq	ERR6363277
*Genome assembly*	
Assembly accession	GCA_911387765.1
*Accession of alternate haplotype*	GCA_911387725.2
Span (Mb)	725
Number of contigs	41
Contig N50 length (Mb)	26.0
Number of scaffolds	36
Scaffold N50 length (Mb)	27.0
Longest scaffold (Mb)	33.2
BUSCO * genome score	C:98.8%[S:98.3%,D:0.4%],F:0.2%,M:1.0%,n:5286
*Genome annotation*	
Number of protein-coding genes	12,096
Average length of coding sequence (bp)	1,534.63
Average number of exons per transcript	7.83
Average exon size (bp)	207.85
Average intron size (bp)	2,914.74

*BUSCO scores based on the lepidoptera_odb10 BUSCO set using v5.1.2. C= complete [S= single copy, D=duplicated], F=fragmented, M=missing, n=number of orthologues in comparison. A full set of BUSCO scores is available at https://blobtoolkit.genomehubs.org/view/ilPyrMalv3.1/dataset/CAJVQT01/busco.

**Figure 3.**
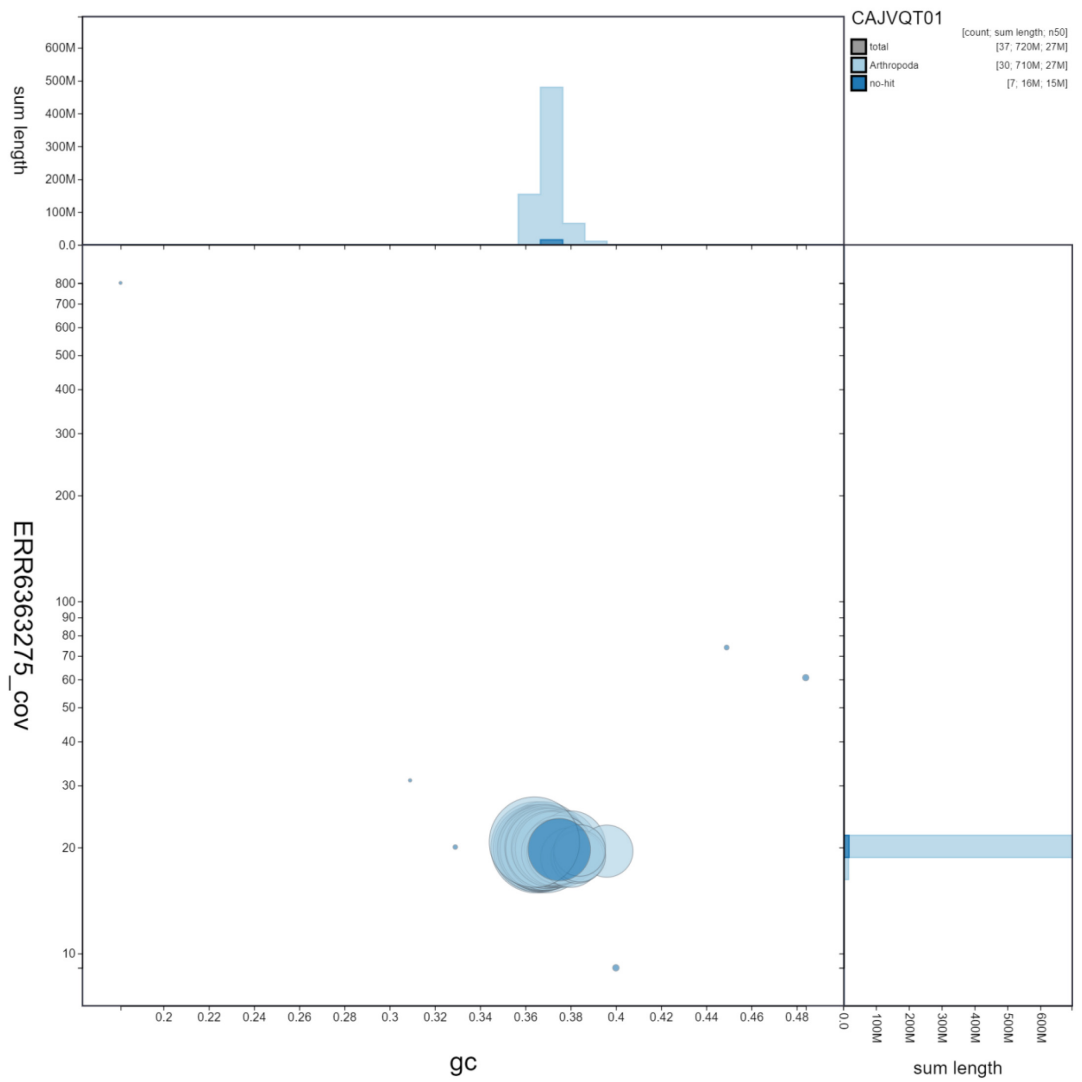
Genome assembly of *Pyrgus malvae*, ilPyrMalv3.1: GC coverage. BlobToolKit GC-coverage plot. Scaffolds are coloured by phylum. Circles are sized in proportion to scaffold length. Histograms show the distribution of scaffold length sum along each axis. An interactive version of this figure is available at https://blobtoolkit.genomehubs.org/view/ilPyrMalv3.1/dataset/CAJVQT01/blob.

**Figure 4.**
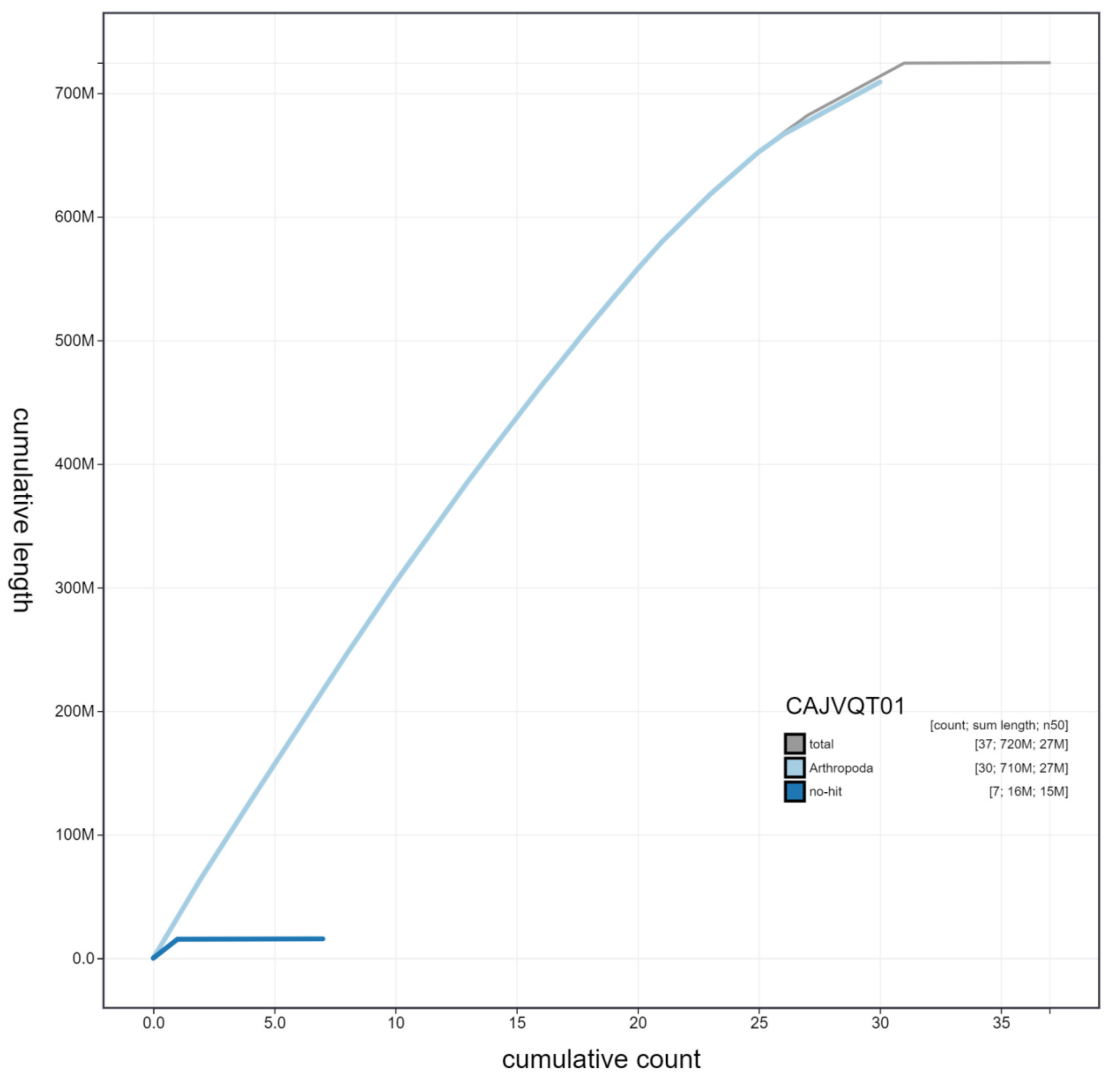
Genome assembly of *Pyrgus malvae*, ilPyrMalv3.1: cumulative sequence. BlobToolKit cumulative sequence plot. The grey line shows cumulative length for all scaffolds. Coloured lines show cumulative lengths of scaffolds assigned to each phylum using the buscogenes taxrule. An interactive version of this figure is available at https://blobtoolkit.genomehubs.org/view/ilPyrMalv3.1/dataset/CAJVQT01/cumulative.

**Figure 5.**
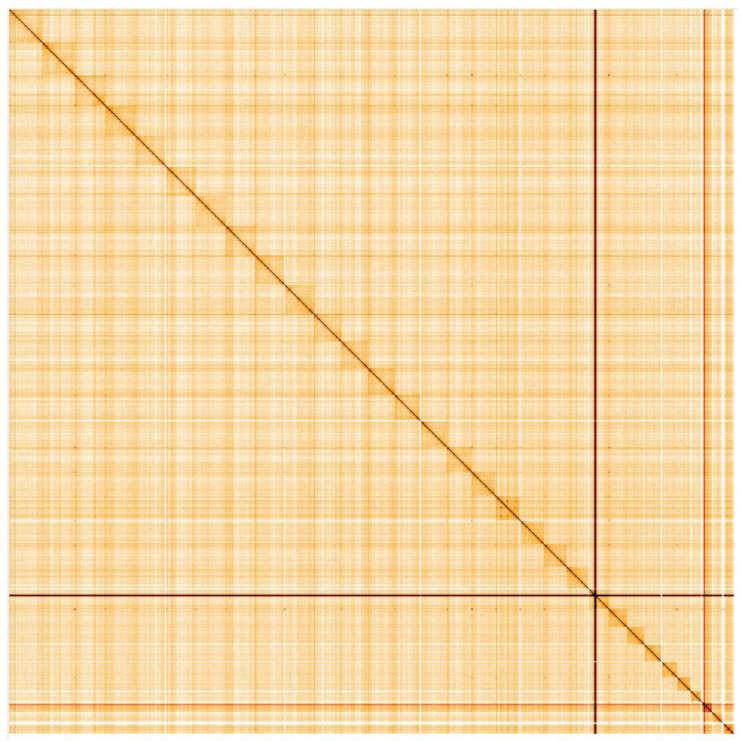
Genome assembly of *Pyrgus malvae*, ilPyrMalv3.1: Hi-C contact map. Hi-C contact map of the ilPyrMalv3.1 assembly, visualised in HiGlass. Chromosomes are shown in size order from left to right and top to bottom. The interactive Hi-C map can be viewed here.

**Table 2.** Chromosomal pseudomolecules in the genome assembly of *Pyrgus malvae*, ilPyrMalv3.1.

INSDC accession	Chromosome	Size (Mb)	GC%
OU426946.1	1	33.22	36.5
OU426948.1	2	30.61	36.9
OU426949.1	3	30.48	36.8
OU426950.1	4	30.18	36.6
OU426951.1	5	30.06	36.5
OU426952.1	6	29.97	36.8
OU426953.1	7	29.61	36.7
OU426954.1	8	29.23	36.7
OU426955.1	9	28.53	36.8
OU426956.1	10	27.64	36.6
OU426957.1	11	27.02	36.7
OU426958.1	12	26.98	36.9
OU426959.1	13	25.95	36.9
OU426960.1	14	25.59	36.9
OU426961.1	15	25.29	36.9
OU426962.1	16	24.25	37.1
OU426963.1	17	24.17	37.5
OU426964.1	18	23.61	37.2
OU426965.1	19	22.98	37.1
OU426966.1	20	21.93	37.2
OU426967.1	21	19.36	38.0
OU426968.1	22	19.17	37.5
OU426969.1	23	17.50	37.3
OU426970.1	24	16.66	37.5
OU426971.1	25	15.31	37.5
OU426972.1	26	14.10	38.0
OU426974.1	27	10.67	39.6
OU426973.1	28	10.82	38.3
OU426975.1	29	10.54	38.4
OU426976.1	30	10.42	38.4
OU426947.1	Z	32.47	36.4
OU426977.1	MT	0.02	18.4
-	Unplaced	0.34	41.9

## Genome annotation report

The ilPyrMalv3.1 genome has been annotated using the Ensembl rapid annotation pipeline ( Table 1; https://rapid.ensembl.org/Pyrgus_malvae_GCA_911387765.1/). The resulting annotation includes 23,484 transcribed mRNAs from 12,096 protein-coding and 2,976 non-coding genes. There are 1.66 coding transcripts per gene and 7.83 exons per transcript.

## Methods

### Sample acquisition and nucleic acid extraction

A male *P. malvae* specimen (ilPyrMalv3, male, genome assembly) was collected from Suatu, Cluj County, Romania (latitude 46.7648, longitude 23.9845) using a net by Konrad Lohse, Alex Hayward Dominik Laetsch and Roger Vila, who also identified the sample. A further two specimens (ilPyrMalv2, unknown sex, Hi-C; ilPyrMalv1, RNA-Seq) were collected from Baciu, Cluj County, Romania (latitude 46.8, longitude 23.5) using a net and were identified by the same team. All samples were snap-frozen at -80°C.

DNA was extracted from the whole organism of ilPyrMalv3 at the Wellcome Sanger Institute (WSI) Scientific Operations core from the whole organism using the Qiagen MagAttract HMW DNA kit, according to the manufacturer’s instructions. RNA (from the whole organism of ilPyrMalv1) was extracted in the Tree of Life Laboratory at the WSI using TRIzol, according to the manufacturer’s instructions. RNA was then eluted in 50 μl RNAse-free water and its concentration RNA assessed using a Nanodrop spectrophotometer and Qubit Fluorometer using the Qubit RNA Broad-Range (BR) Assay kit. Analysis of the integrity of the RNA was done using Agilent RNA 6000 Pico Kit and Eukaryotic Total RNA assay.

### Sequencing

Pacific Biosciences HiFi circular consensus and 10X Genomics read cloud DNA sequencing libraries were constructed according to the manufacturers’ instructions. Poly(A) RNA-Seq libraries were constructed using the NEB Ultra II RNA Library Prep kit. DNA and RNA sequencing was performed by the Scientific Operations core at the WSI on Pacific Biosciences SEQUEL II (HiFi), Illumina HiSeq X (10X) and Illumina HiSeq 4000 (RNA-Seq) instruments. Hi-C data were also generated from whole organism tissue of ilPyrMalv2 using the Arima v1 Hi-C kit and sequenced on an Illumina NovaSeq 6000 instrument.

### Genome assembly

Assembly was carried out with Hifiasm ( Cheng *et al.*, 2021); haplotypic duplication was identified and removed with purge_dups ( Guan *et al.*, 2020). One round of polishing was performed by aligning 10X Genomics read data to the assembly with longranger align, calling variants with freebayes ( Garrison & Marth, 2012). The assembly was then scaffolded with Hi-C data ( Rao *et al.*, 2014) using SALSA2 ( Ghurye *et al.*, 2019). The assembly was checked for contamination and corrected using the gEVAL system ( Chow *et al.*, 2016) as described previously ( Howe *et al.*, 2021). Manual curation ( Howe *et al.*, 2021) was performed using gEVAL, HiGlass ( Kerpedjiev *et al.*, 2018) and Pretext. The mitochondrial genome was assembled using MitoHiFi ( Uliano-Silva *et al.*, 2021), which performed annotation using MitoFinder ( Allio *et al.*, 2020). The genome was analysed and BUSCO scores generated within the BlobToolKit environment ( Challis *et al.*, 2020). Table 3 contains a list of all software tool versions used, where appropriate.

**Table 3.** Software tools used.

Software tool	Version	Source
Hifiasm	0.15.1	Cheng *et al.*, 2021
purge_dups	1.2.3	Guan *et al.*, 2020
SALSA2	2.2	Ghurye *et al.*, 2019
longranger align	2.2.2	https://support.10xgenomics.com/genome-exome/software/pipelines/latest/advanced/other-pipelines
freebayes	1.3.1-17-gaa2ace8	Garrison & Marth, 2012
MitoHiFi	2	Uliano-Silva *et al.*, 2021
gEVAL	N/A	Chow *et al.*, 2016
HiGlass	1.11.6	Kerpedjiev *et al.*, 2018
PretextView	0.2.x	https://github.com/wtsi-hpag/PretextView
BlobToolKit	2.6.4	Challis *et al.*, 2020

### Genome annotation

The Ensembl gene annotation system ( Aken *et al.*, 2016) was used to generate annotation for the *Pyrgus malvae* assembly ( GCA_911387765.1). Annotation was created primarily through alignment of transcriptomic data to the genome, with gap filling via protein-to-genome alignments of a select set of proteins from UniProt ( UniProt Consortium, 2019).

## Data availability

European Nucleotide Archive: Pyrgus malvae (grizzled skipper). Accession number PRJEB45665; https://identifiers.org/ena.embl/PRJEB45665.

The genome sequence is released openly for reuse. The *P. malvae* genome sequencing initiative is part of the Darwin Tree of Life (DToL) project. All raw sequence data and the assembly have been deposited in INSDC databases. Raw data and assembly accession identifiers are reported in Table 1.

## Author information

Members of the Darwin Tree of Life Barcoding collective are listed here: https://doi.org/10.5281/zenodo.5744972.

Members of the Wellcome Sanger Institute Tree of Life programme are listed here: https://doi.org/10.5281/zenodo.6125027.

Members of Wellcome Sanger Institute Scientific Operations: DNA Pipelines collective are listed here: https://doi.org/10.5281/zenodo.5746904.

Members of the Tree of Life Core Informatics collective are listed here: https://doi.org/10.5281/zenodo.6125046.

Members of the Darwin Tree of Life Consortium are listed here: https://doi.org/10.5281/zenodo.5638618.
