# Supplementary material for: The genome sequence of the grizzled skipper, Pyrgus malvae (Linnaeus, 1758)
Source: Wellcome Open Res. 2022 Mar 29;7:114. [Version 1] doi: 10.12688/wellcomeopenres.17806.1 (PMC9156896; doi:10.12688/wellcomeopenres.17806.1)
Supplement: Peer review history

# Peer review: Reviewer response for version 1

**DOI:** 10.21956/wellcomeopenres.19712.r49569

**Published:** 2022-05-31

**Authors:** Pável Matos-Maraví

**Affiliations:** 1 Biology Centre of the Czech Academy of Sciences, Institute of Entomology, České Budějovice, České Budějovice, Czech Republic

Hayward *et al.* report the genome sequence of *Pyrgus malvae*. The article describes well the relevance of having an annotated reference genome for the species. The methods are clearly described and allow the reproducibility of the experiments. Perhaps of interest for people interested in generating further reference genomes of Hesperiidae, it would be informative to state the amount of high molecular weight DNA used for each HiFi circular consensus and 10X Genomics read cloud sequencing libraries.

 The authors ensured that the data is open and that the figures and tables describe the output in a very informative way.

Are sufficient details of methods and materials provided to allow replication by others?

Yes

Is the rationale for creating the dataset(s) clearly described?

Yes

Are the datasets clearly presented in a useable and accessible format?

Yes

Are the protocols appropriate and is the work technically sound?

Yes

Reviewer Expertise:

Lepidoptera, Phylogenetics, Macroevolution

I confirm that I have read this submission and believe that I have an appropriate level of expertise to confirm that it is of an acceptable scientific standard.

# Peer review: Reviewer response for version 1

**DOI:** 10.21956/wellcomeopenres.19712.r49571

**Published:** 2022-04-21

**Authors:** Marko Mutanen

**Affiliations:** 1 Ecology and Genetics Research Unit, University of Oulu, Oulu, Finland

This work published a genome for a butterfly species representing family Hesperiidae. High-quality genomes like the one generated here are of great value for people working on genomics of butterflies and other taxa. I was carefully reading the manuscript through and found it very well prepared. As far as I can see, there are no questions concerning the validity of the methods and conducted analyses, and the quality of this work appears very good to me. I am, however, not very familiar with several of the analytical tools used in this study. Despite this, I am fully confident that this data merits being published in Wellcome Open Research journal and that there are no major (nor even minor) flaws in this paper. I am therefore happy to recommend its publication in its present form with two very minor reservations:

1. In the Genome annotation report section, the authors state that 2,976 non-coding genes were annotated. What are non-coding genes given that by definition genes are coding regions of genome?
2. I think a comma is lacking after the name Hayward on page 3.

Are sufficient details of methods and materials provided to allow replication by others?

Yes

Is the rationale for creating the dataset(s) clearly described?

Yes

Are the datasets clearly presented in a useable and accessible format?

Yes

Are the protocols appropriate and is the work technically sound?

Yes

Reviewer Expertise:

Biodiversity genomics, DNA barcoding, Insect taxonomy

I confirm that I have read this submission and believe that I have an appropriate level of expertise to confirm that it is of an acceptable scientific standard.

# Peer review: Reviewer response for version 1

**DOI:** 10.21956/wellcomeopenres.19712.r49570

**Published:** 2022-04-20

**Authors:** Jeffrey M. Marcus

**Affiliations:** 1 Department of Biological Sciences, University of Manitoba, Winnipeg, MB, Canada

This manuscript describes the sequencing and genome assembly of the European grizzled skipper *Pyrgus malvae*. There are some conservation concerns in parts of the range of this species, making collecting its genome sequence justifiable. The work described is competent and the writing is clear. Most of the data is clearly presented in a useable and accessible format. The one exception is that it is not clear where to find the assembled sequences for the second haplotype. I can find only one set of accessions in the text of the manuscript, presumably for the first haplotype. Where the second haplotype can be found should be made clear.

**Minor Concerns:**

- p. 3 Background: First 4 sentences of the background are without in-text citations. Where does this information come from? 
- p. 3Similarly in 3 ^rd^ background paragraph, no citations for:“When fully-grown, the larva constructs a cocoon at the base of low vegetation, where it overwinters as a pupa. Adults feed on a wide variety of nectar sources, including Bird’s foot trefoil ( *Lotus corniculatus*), bugle ( *Ajuga reptans*), buttercup ( *Ranunculus *species), daisy ( *Bellis perennis*), and dandelion ( *Taraxacum officinale*).”
- p. 3 suggested reword “At the European level this species is listed as Least Concern in the IUCN Red List…”.
- p. 3 “While not fully phased, the assembly deposited is of one haplotype. Contigs corresponding to the second haplotype have also been deposited.” Where is the second haplotype deposited?  I can only find set of INSDC accession numbers in Table 2, which I presume are associated with the first haplotype.

Are sufficient details of methods and materials provided to allow replication by others?

Yes

Is the rationale for creating the dataset(s) clearly described?

Yes

Are the datasets clearly presented in a useable and accessible format?

Partly

Are the protocols appropriate and is the work technically sound?

Yes

Reviewer Expertise:

genomics, Lepidoptera, phylogenetics, genetics, evo-devo

I confirm that I have read this submission and believe that I have an appropriate level of expertise to confirm that it is of an acceptable scientific standard, however I have significant reservations, as outlined above.
